# The MRTF-A/miR-155/SOX1 pathway mediates gastric cancer migration and invasion

**DOI:** 10.1186/s12935-020-01395-5

**Published:** 2020-07-11

**Authors:** Libin Yin, Tao Liu, Chenyao Li, Guoqiang Yan, Chao Li, Jiantao Zhang, Lei Wang

**Affiliations:** Department of Colorectal and Anal Surgery, The First Hospital of Jilin University, Changchun, Jilin, 130021 China

**Keywords:** Gastric cancer, MRTF-A, miR-155, SOX1, Migration

## Abstract

**Background:**

Gastric cancer (GC) is the leading cause of death worldwide and is closely related to metastasis. MRTF-A is one of the most well-characterized genetic markers in cancer. However, the mechanism whereby MRTF-A mediate gastric cancer (GC) tumorigenesis is not fully clear. Increasing evidence has confirmed that miRNA dysregulation is involved in MRTF-A-mediated tumorigenesis, supporting their potential as therapeutic targets for cancer. Although miR-155 has been reported as an upregulated miRNA, the interplay between miR-155 and MRTF-A-mediated gastric cancer progression remain largely elusive.

**Methods:**

Real-time PCR was performed to determine miR-155 expression after transfected with MRTF-A encoding plasmids and siRNA. Potential target genes were identified by Western blot and luciferase reporter assay. Chip assay was proved that MRTF-A binds in the promoter region of miR-155. Transwell assay and Scratch-healing migration assay was used to investigate the role of MRTF-A and SOX1 in gastric cancer cell migration and invasion.

**Results:**

MRTF-A can interact with the miR-155 promoter to promote histone acetylation and RNA polymerase II recruitment via the Wnt-β-catenin pathway. miR-155 promotes gastric cancer cell migration by suppressing SOX1 expressiom by targeting its 3′UTR in vitro and in vivo. MRTF-A inhibited the inhibitory effects of SOX1 on gastric cancer cell migration by promoting the express -ion of miR-155.

**Conclusion:**

Our data therefore provide important and novel insights into how the MRTF-A/miR-155/SOX1 pathway mediates migration and invasion in GC.

## Background

Gastric cancer (GC) remains one of the most common causes of cancer-related deaths worldwide and is the fourth leading cause of tumor-related death in the world [1]. Despite the substantial improvements in chemotherapy, radiotherapy and surgical techniques for gastric cancer over the past few decades, the prospect for patients with gastric cancer is not optimistic [2]. Metastasis is a complex pathophysiological process, beginning with the migration and invasion of cancer cells into the surrounding tissues and lymph system [3]. Cell migration is associated with multiple signaling pathways and transcription factors and plays a critical role in many biological processes [4]. Therefore, it is necessary to acquire a better understanding of the targeted molecules involved in gastric cancer, as well as improve early detection and identify more effective treatments.

MicroRNAs (miRNAs) are a class of endogenous, small oncoding RNAs that are approximately 20–25 nucleotides in length that can regulate gene expression [5]. MiRNAs, a group of oncoding RNAs that specifically bind to the mRNA 3′-untranslated region of target genes, negatively regulate gene expression at the post-transcriptional level [6]. MiRNAs play an important role in cell growth, movement, invasion and stress responses [7, 8]. Therefore, it is necessary to acquire a better understanding of miRNA-regulated molecular pathways in controlling gastric cancer, as well as develop novel targeting strategies for the treatment of gastric cancer. miR-155 promotes gastric cancer initiation and metastasis [9]. It has been shown that miR-155 promotes cell growth and migration by negatively regulating TGFβR2. miR-155 decreases TGFβR2 to regulate gastric cancer cell proliferation by interacting with target sites in the TGFβR2 3′ untranslated region (3′UTR) [10].

MRTF-A (also named MKL1) plays a key role in cell proliferation, differentiation, migration, and apoptosis [11]. MRTF-A, is a coactivator of SRF (serum response factor), that is expressed in a wide range of tissues [12]. In nuclei, the MRTF-A-SRF complex binds to the CArG-box on target promoters to activate transcription [13–17]. The Y (SRY)-box (SOX) protein family is a group of transcription factors containing a highly conserved high mobility group (HMG) DNA binding domain [14]. SOX family members play crucial roles in both embryonic and postnatal development as well as in stem cell regulation [15–17]. Moreover, several members of the SOX family have been implicated in cancer development [18–23]. For example, SOX1 is a tumor suppressor in cervical and ovarian cancers. SOX1 was detected in advanced stages of ESCC progression, as well as in highly invasive and aggressive tumor tissues. SOX10 contributes to tumorigenesis in hepatocellular carcinoma (HCC). SOX1 inhibits breast cancer cell growth and invasion by suppressing the Wnt/β-catenin signaling pathway.

In the present study, we demonstrated that MRTF-A physically interacts with the miR-155 promoter to promote histone acetylation and RNA polymerase II recruitment through the Wnt-β-catenin pathway. miR-155 promotes the migration of gastric cancer cells by suppressing the expression of SOX1by targeting its 3′UTR in vitro and in vivo. The identification of the MRTF-A/miR-155/SOX1signaling pathway provides new insights for the occurrence and development of gastric cancer.

## Materials and methods

### Cell culture and transfection

Human gastric cancer SGC-7901 and MGC-803 cells were cultured in RPMI-1640 medium (Thermo Fisher Scientific, Inc., Waltham, MA, USA) supplemented with 10% fetal bovine serum (Sigma-Aldrich; Merck KGaA, Darmstadt, Germany) at 37 °C in a humidified incubator with 5% CO_2_. miR-155 mimics, inhibitor, si-MRTF-A, si-SRF, si-SOX1 and the scrambled negative control were purchased from Ribobio. (Guangzhou, China). Cells were seeded in 6-well plates, incubated overnight, and then transiently transfected with miR-155 mimics, miR-negative control of mimics (miR-Ctrl), miR-155 inhibitor or miR-negative control of inhibitor (anti-miR-Ctrl) using Lipofectamine 2000 (Invitrogen, Inc., Waltham, MA, USA) according to the manufacturer’s instructions. The full length MRTF-A cDNA (which included the ORF and 3′UTR) was PCR-amplified and cloned into the pcDNA3.1 vector to generate the pcDNA3.1-MRTF-A construct that was used in the assays. siMRTF-A targets human MRTF-A at 5′-ATCACGTGTGATTGACATGTA-3′, siSRF targets human SRF at 5′-CAAGATGGAGTTCATCGACAA-3′. and siSOX1 targets human SOX1 at 5′-AGAGGAAGGCTTGGGAGTA-3′. As a control, we used the negative-control siRNA from Ribobio.

### Extraction of total RNA and RT-qPCR

Total RNA was extracted with the Trizol reagent (Invitrogen). cDNA was synthesized with M-MLV reverse transcriptase (Promega) and quantified by realtime qPCR using a Biosystems StepOne™ Real-Time PCR system and Fast SYBR Green Master Mix (Applied Biosystems), with GAPDH and U6 as internal controls. PCR primers were designed with the NCBI online software Primer-BLAST and synthesized by Invitrogen. The PCR conditions were as follows: 94 °C for 2 min, followed by 30 cycles of 94 °C for 30 s, 60 °C for 30 s and 72 °C for 1 min, and 72 °C for 10 min. The primer sequences were as follows:U6,5′-GATTACAGCCGAACGTGTAGGA A-3′(forward); and 5′-AGCTTG ATCGTTTCTCTGGCCACC-3′(reverse);GAPDH, 5′-TCCAGAGTGCAAGGCTTCAG-3′(forward); and 5′-ACAGCACGCAGTAGCA GTA-3′ (reverse); MRTF-A, 5′-TGTGTCATCTTGACTCGCCT-3′ (forward); and 5′-TCATGAAATGCGGCTG GACT-3′ (reverse);SRF,5′-AGTCCTCAACGCTTTC TCG-3′ (forward); and; 5′-G CTGCCACTGCTGCTCT-3′ (reverse); miR-155, 5′-AC GCTCAGTTAATGCTAAT CGTGA-3′ (forward); and; 5′-ATTCCATGTTGTCCA CTGTCTCTG-3′ (reverse); SOX1,5′-AGAGGAAGGCTTGGGAGTA-3′ (forward); and; 5′-GGGCAGCAGAG CTATGTG-3′ (reverse);

### Western-blotting

Proteins in cell lysates were separated by SDS-PAGE and then transferred to a nitrocellulose membrane (Millipore). The primary antibodies included SOX1 (Santa Cruz Biotechnology), MRTF-A (Santa Cruz Biotechnology), SRF (Santa Cruz Biotechnology), and GAPDH (Santa Cruz Biotechnology). The corresponding primary antibodies were used according to the manufacturer’s instructions, followed by incubation with an HRP-conjugated secondary antibody (Sigma-Aldrich, USA). The LabWorks Image Acquisition and Analysis Software (UVP, Upland, CA, USA) was used to measure the band density.

### Chromatin immunoprecipitation (ChIP)

ChIP assays were carried out following a standard protocol. Briefly, SGC-7901 cells were cross-linked with 1% formaldehyde (Sigma) for 15 min at room temperature with gentle shaking. Cells were harvested using a silicon scraper and flash frozen in liquid nitrogen and stored at − 80 °C prior to use. Cells were resuspended, lysed in lysis buffers, and sonicated to solubilize and shear crosslinked DNA. Sonication conditions vary depending on cells, culture conditions, crosslinking, and equipment. We used a Misonix Sonicator 3000 and sonicated at power 6 for 10 × 40 s pulses (60 s pause between pulses) at 4 °C while samples were immersed in an ice bath. The cells were fragmented to an average of 300 bp in length with a sonicator. Diluted chromatin sonicates were incubated with anti-MRTF-A (Santa Cruz Biotechnology), anti-RNAPII (Abcam) or anti-AcH3K9 (Abcam) antibodies. The resulting whole-cell extract was incubated overnight at 4 °C with 100 μl of protein A beads (Millipore) that had been preincubated with 10 μg of the appropriate antibody. Beads were washed four times with RIPA buffer and two times with TE containing 50 mM NaCl. Bound complexes were eluted from the beads by heating at 65 °C with occasional vortexing for 30 min. crosslinking was reversed and protein digestion by Proteinase-K overnight incubation at 65 °C. Immunoprecipitated DNA and whole-cell extract DNA were then purified by treatment with multiple phenol, chloroform and isopropanol extractions. After washing and eluting, the ChIP products were purified and measured by real-time qPCR. The primers used for the amplification of the hsa-miR-155 promoter region between − 503 to − 317 bp were as follows: hsa-miR- 155-CArG-box, forward 5′-ATTTTGAACATTTGGGC-3′, and reverse, 5′-TCTAGTTTTGAATCGGG-3′.

### Scratch-healing migration assay

Approximately 5 × 10^4^ cells were seeded in 24-well plates and cultured to 70–80% confluence. Wounds were established using a 20-μl pipette tip and the cells were allowed 24 h to migrate into the wounds. To assess the cell migrations across the artificial wound, five optical fields were randomly selected. Images were acquired with a microscope (Zeiss, Axiovert 200, Germany).

### Cell invasion assay

For the invasion assay, the membranes were used and precoated with Matrigel (2 μg/well; BD Biosciences, San Jose, CA, USA) to simulate a matrix barrier. Cells in each group were digested with 0.25% trypsin and centrifuged before diluting with RPMI-1640 culture medium to 5 × 10^5^/ml. Diluted SGC-7901 cells were seeded onto the upper chamber of a Transwell insert coated with Matrigel matrix and 0.6 mL RPMI-1640 medium containing 20% FBS was added into the lower chamber before culturing in an incubator at 37 °C and 5% CO_2_ for 24 h. After the experiment, 4% paraformaldehyde was used to fix the cell membrane and cotton swabs were used to wipe cells whose upper surface did not penetrate. The number of cells penetrating the Matrigel was recorded as the number of invasive cells.

### Luciferase reporter assay

miR-155(− 450 to − 390, transcription initiation site designated as + 1) was cloned into the promoterless pGL3-basic vector (Promega) to prepare the luciferase reporter plasmid (Wt). A reporter plasmid containing the mutant miR-155 promoter (Mut) was constructed by mutating the wild-type CArG (CATTTTGG to AATTTTGG). Cells were transfected with the pGL3- basic vector (Promega) containing the promoter variants, MRTF-A overexpression plasmid and siRNA for 24 h. The human SOX1 gene 3′untranslated region (3′UTR) was cloned into the promoterless pGL3- basic vector (Promega) to prepare the luciferase reporter plasmid. Cells were transfected with the pGL3-basic vector (Promega) containing the 3′UTR variants miR-155 mimics and inhibitor for 24 h. After transfection of the luciferase plasmids, luciferase activity was measured with a Luciferase Assay System (Promega) in a Synergy™ 4 Luminometer (BioTek).

### In vivo tumorigenicity assay

Male BALB/c nude mice aged 4–5 weeks were purchased from BEIJING HFK BIOSCIENCE CO.,LTD. The mice were housed in a pathogen-free animal facility and randomly assigned to the control or experimental group (five mice per group). For each cell line, 2 × 10^6^ cells were resuspended in 200 μl of medium and then subcutaneously injected into the nude mice. Tumor formation was monitored every 3 or 4 days by measuring the largest and smallest diameter of the formed tumors. At euthanasia, the tumors were recovered and the wet weights of each tumor were examined. Animal care practices and all experiments were reviewed and approved by the Committee on the Ethics of Animal Experiments of the First Hospital of Jilin University.

### Immunohistochemistry

Immunohistochemical analysis was performed as described previously. Briefly, tumor sections were incubated overnight in the primary SOX1 antibody (1:50) in conjunction with proper controls. The sections were then washed three times with 0.05% Tween, incubated with secondary antibody for 1 h, washed three times with 0.05% Tween in PBS, visualized with the 3,3′-diaminobenzidine (DAB) substrateand counterstained with hematoxylin QS (Vector Lab, Burlingame, CA, USA).

### Statistical analysis

All statistical analyses were performed using SPSS 17.0 (SPSS, Chicago, USA) with either a one-sample *t* test or one-way ANOVA. All data are presented as the mean ± S.E.M. A *p* value less than 0.05 is indicated with an *, and a value less than 0.01 is indicated with a **.

## Results

### MRTF-A played a positive role in the regulation of miR-155 expression in GC cells

**Fig. 1:**
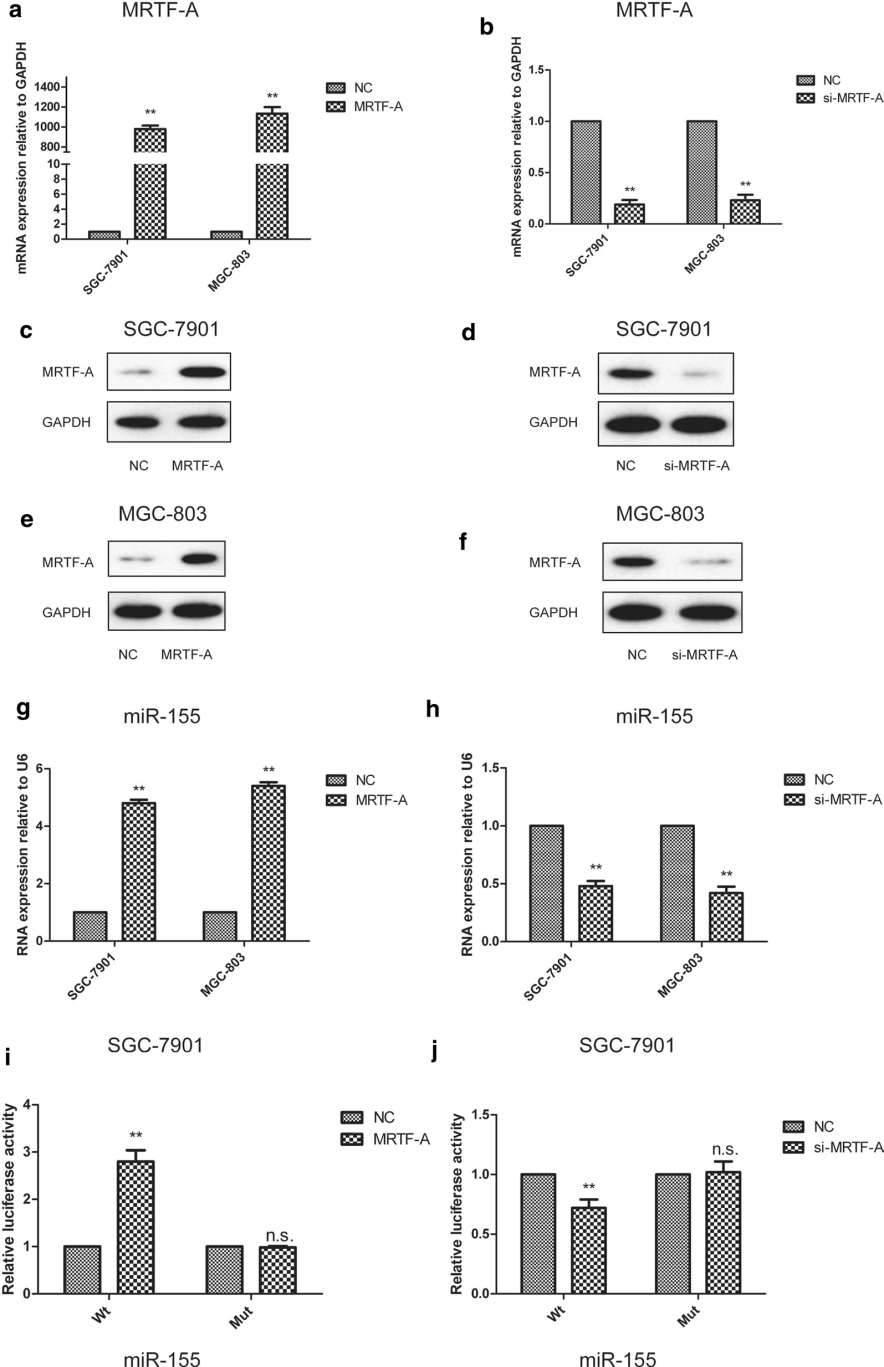
MRTF-A has a positive role in the regulation of miR-155 expression in GC cells. MRTF-A over-expression upregulated miR-155 and MRTF-A RNA levels in SGC-7901 and MGC-803 cells (**a**, **g**). MRTF-A over-expression increased MRTF-A protein levels in SGC-7901 and MGC-803 cells (**c**, **e**). **a**, **c**, **e**, **g** MRTF-A encoding plasmids were transfected into gastric cancer cells; pcDNA3.1 was the vector control. Forty-eight hours after transfection, mRNA levels were determined by RT-qPCR and protein levels were determined by Western-blot. GAPDH was used as a loading control. MRTF-A depletion with siRNA reduced miR-155 and MRTF-A RNA levels in SGC-7901 and MGC-803 cells (**b**, **h**). MRTF-A depletion with siRNA suppressed MRTF-A protein expression in SGC-7901 and MGC-803 cells (**d**, **f**). **b**, **d**, **f**, **h** Cells were transfected with siMRTF-A duplexes. mRNA and protein levels were determined 48 h after transfection. **i** MRTF-A over-expression elevated miR-155 promoter activity in SGC-7901 gastric cancer cells. **j** siMRTF-A reduced miR-155 promoter activity in SGC-7901 gastric cancer cells. Data are shown as the mean ± S.E.M, based on three to six independent experiments. **, p < 0.01

MRTF-A has previously been shown to promote the expression of miR-155 in cancer [24]. To investigate the effect of MRTF-A on miR-155 in gastric cancer cells, the MRTF-A gene was cloned. After overexpression of MRTF-A, miR-155 expression level were significantly increased in SGC-7901 and MGC-803 gastric cancer cells (Fig. 1a, c, e, g). To confirm the role of MRTF-A in miR-155 gene regulation, MRTF-A was knocked down with si-MRTF-A. Deletion of MRTF-A resulted in a significant decrease in miR-155 RNA levels (Fig. 1b, d, f, h). Luciferase assay results showed that miR-155 promoter activity was significantly increased following MRTF-A overexpression in SGC-7901 cells (Fig. 1i). Furthermore, MRTF-A deletion inhibited the promoter activity of miR-155 (Fig. 1j).

**Fig. 2:**
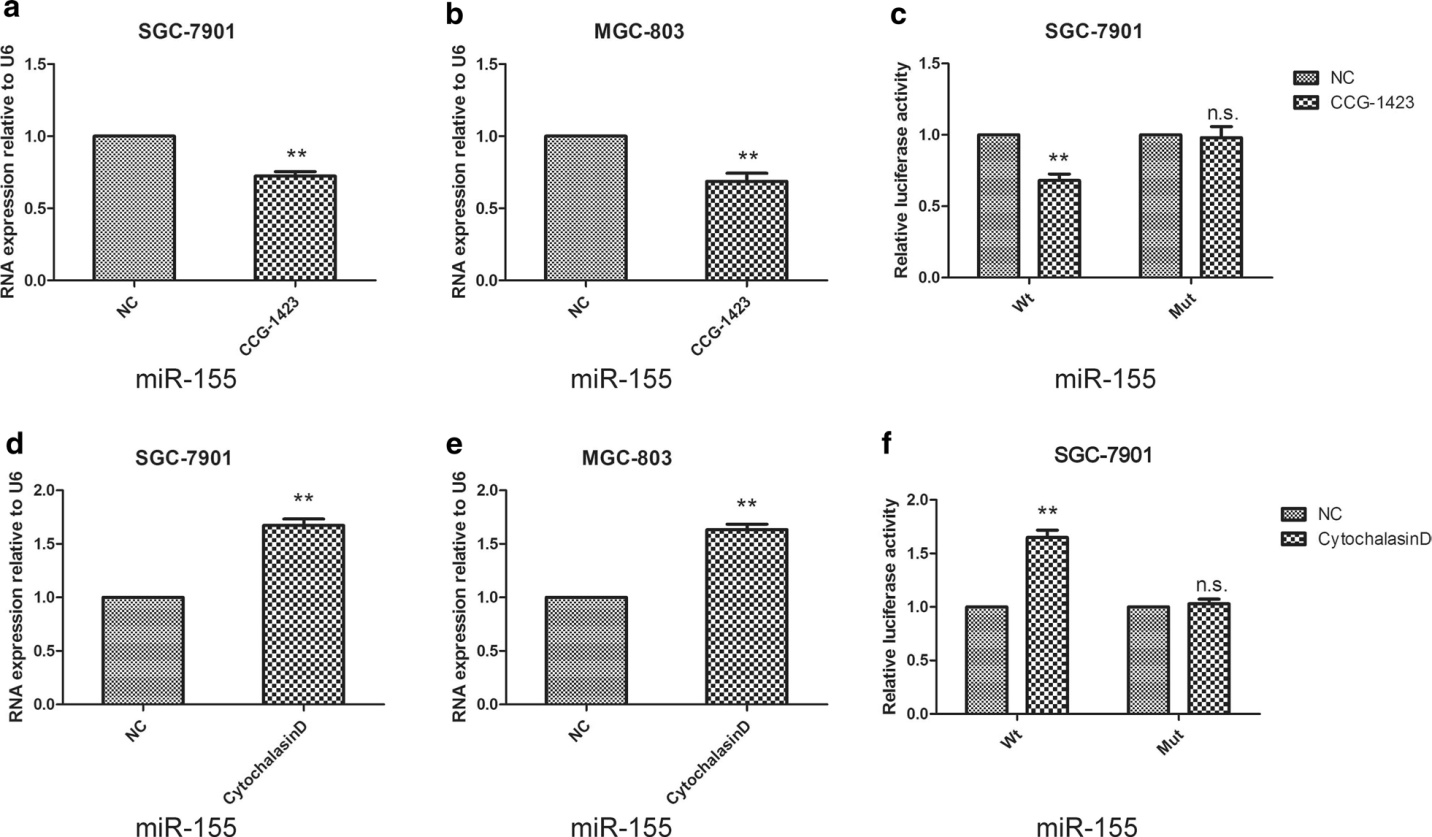
MRTF-A plays a positive role in the regulation of miR-155 expression in GC cells. miR-155 expression was decreased by the selective MRTF-A inhibitor CCG-1423. **a**, **b** CCG-1423 inhibited miR-155 expression in SGC-7901 and MGC-803 cells. **a**–**c** Cells were treated with 10 μM CCG-1423 for 24 h. RNAs were examined by RT-qPCR. **c** CCG-1423 inhibited miR-155 promoter activity in SGC-7901 cells. miR-155 expression was stimulated by Cytochalasin D. **d**, **e** Cytochalasin D stimulated miR-155 expression in SGC-7901 and MGC-803 cells. **d**–**e** Cells were treated with 10 μM Cytochalasin D for 24 h. RNAs were examined by RT-qPCR. **c** Cytochalasin D increased miR-155 promoter activity in SGC-7901 cells. Data are shown as the mean ± S.E.M, based on three to six independent experiments. **, p < 0.01

Gastric cancer cells were then treated with CCG-1423(inhibited nuclear translocation of MRTF-A). The results showed that miR-155 expression and promoter activity were inhibited by CCG-1423 in SGC-7901 and MGC-803 cells (Fig. 2a–c). Nuclear localization of MRTF-A was required for miR-155 transcription. Next gastric cancer cells were treated with Cytochalasin D (a small chemical molecule that stimulates the nuclear localization of MRTF-A). The results showed that Cytochalasin D increased miR-155 expression and promoter activity in SGC-7901 and MGC-803 cells (Fig. 2d–f). These results suggest that MRTF-A can promote miR-155 transcription.

### MRTF-A promoted the transactivity of miR-155 through the CArG box

**Fig. 3:**
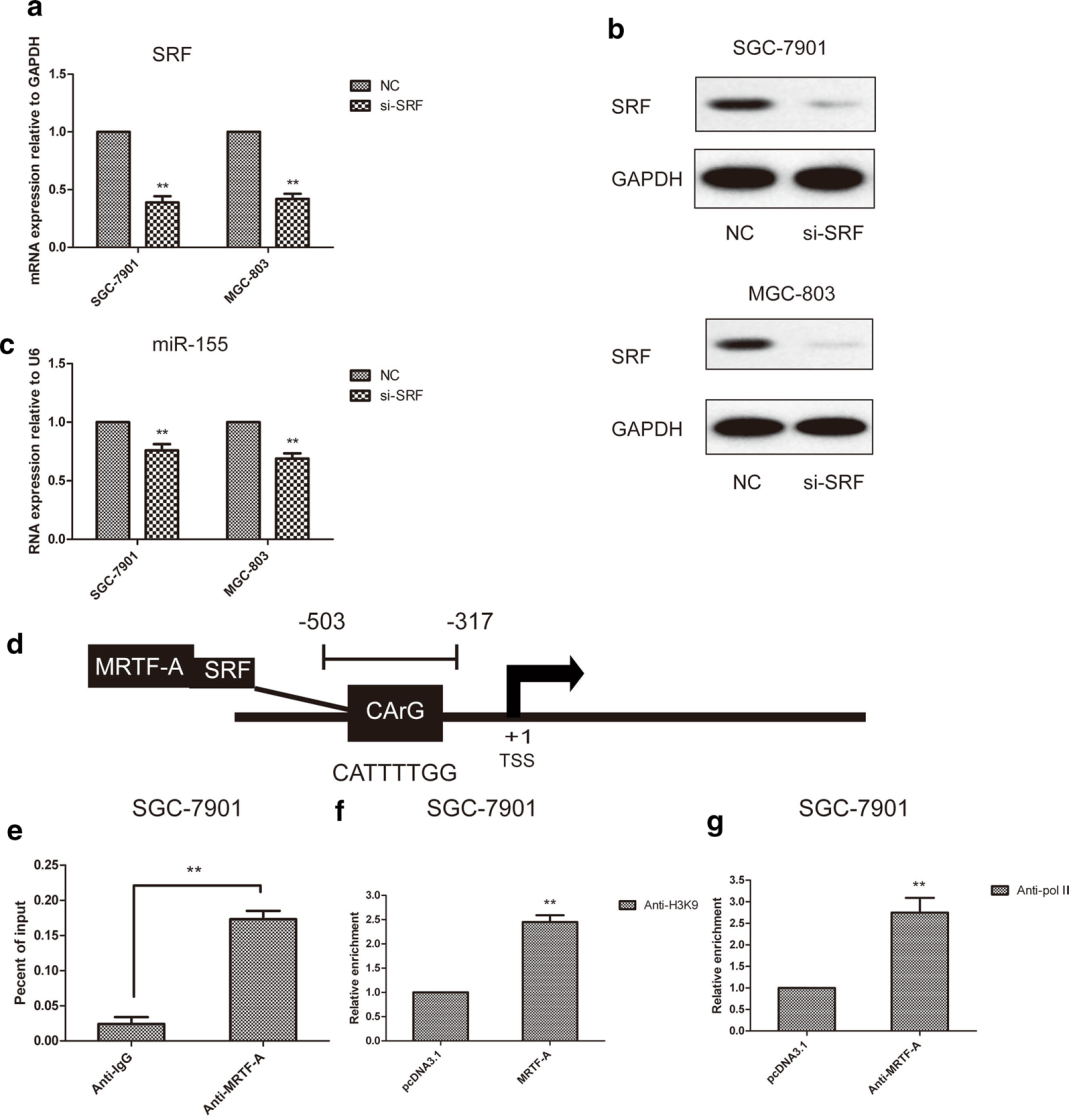
MRTF-A promoted the transactivity of the miR-155 by CArG box (**a**) SRF knockdown with siSRF downregulated SRF mRNA levels in SGC-7901 and MGC-803 cells. **b** SRF protein levels were decreased in SRF depleted SGC-7901 and MGC-803 cells. **c** SRF knockdown with siSRF downregulated miR-155 RNA levels in SGC-7901 and MGC-803 cells. **d** A scheme for the structure of the miR-155 promoter. The position of the CArG-box and the ChIP-qPCR fragment are indicated. **e** MRTF-A was enriched in the CArG-containing promoter of miR-155. ChIP was performed with MRTF-A antibodies; normal rabbit IgG were used as the negative control. ChIP assay products were analyzed by RT-qPCR. **f** Histone acetylation at the miR-155 promoter was increased following MRTF-A overexpression. ChIP was performed with rabbit polyclonal antibodies against acetylated histone H3K9. **g** Overexpression of MRTF-A promoted the recruitment of RNA polymerase II to the miR-155 promoter. Data are shown as the mean ± S.E.M, based on three to six independent experiments. **, p < 0.01

MRTF-A forms a complex with SRF to promote the transcription of target genes and usually binds to the CArG-box site on downstream genes. To examine the role of SRF in miR-155 gene transcription, the SRF gene was knocked down in gastric cancer cells with si-SRF. SRF mRNA and protein levels were significantly reduced in SGC-7901 and MGC-803 gastric cancer cells (Fig. 3a, b). The depletion of SRF resulted in a decrease in miR-155 RNA levels in SGC-7901 and MGC-803 gastric cancer cells (Fig. 3c). This suggests that the MRTF-A-SRF complex promotes miR-155 transcription via association with the CArG-box. ChIP assays were performed tosupport this result. The position of the CArG-box and ChIP-qPCR fragment in the miR-155 promoter is described in Fig. 3d. The results showed that MRTF-A antibodies efficiently precipitated the CArG-containing DNA fragment in the miR-155 promoter, indicating the physical association of MRTF-A with the miR-155 promoter (Fig. 3e). It has been reported that histone H3K9 acetylation can activate transcription [25–27]. Therefore, it is possible that MRTF-A facilitates histone acetylation of the miR-155 promoter. The results showed that acetylated histone H3K9 increased the association with the miR-155 promoter after MRTF-A overexpression (Fig. 3f). Moreover, RNA polymerase II showed increased association with the miR-155 promoter after MRTF-A overexpression (Fig. 3g). These results suggest that MRTF-A plays a direct role in the activation of miR-155.

### MRTF-A was crucial for miR-155 activation induced by Wnt signaling

**Fig. 4:**
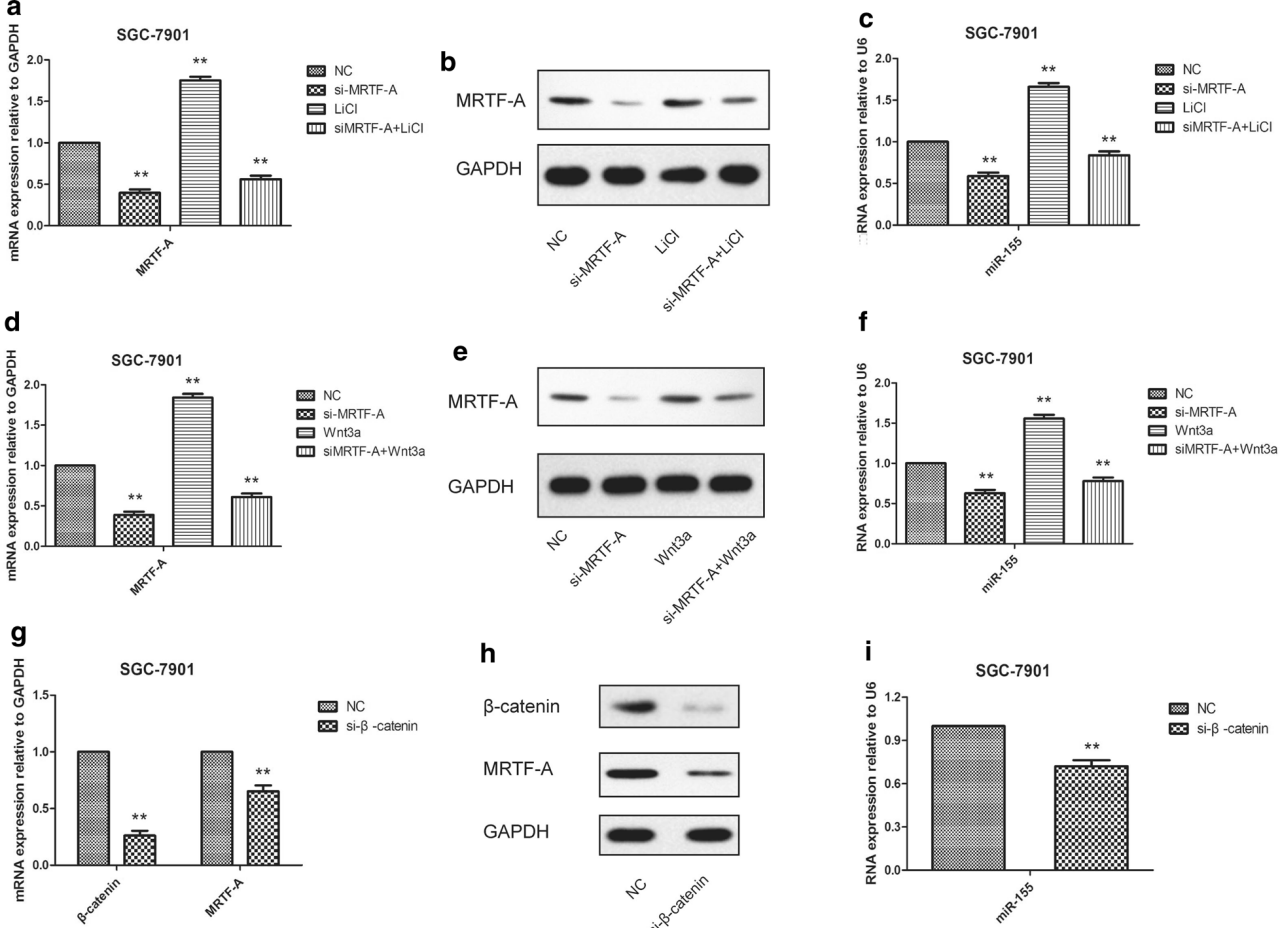
MRTF-A was crucial for miR-155 activation induced by Wnt signaling (**a**, **c**) MRTF-A and miR-155 RNA levels were concurrently upregulated in LiCl-treated SGC-7901cells. **b** MRTF-A protein expression was upregulated in LiCl-treated MCF-7 cells. **a**–**c** Cells were treated with 2.5 mM LiCl for 24 h. Depletion of MRTF-A abolished the LiCl-induced upregulation of miR-155 in SGC-7901 cells. **d**, **f** MRTF-A and miR-155 RNA levels were concurrently increased in Wnt3a-treated SGC-7901 cells. **e** MRTF-A protein expression was concurrently induced in Wnt3a-treated SGC-7901 cells. **d**–**g** Cells were treated with 10 μM Wnt3a for 24 h. Depletion of MRTF-A abolished the Wnt3a-induced upregulation of miR-155 in SGC-7901 cells (**g**, **i**) Knockout of β-catenin concurrently decreased MRTF-A and miR-155 expression in SGC-7901 cells. **h** MRTF-A protein expression was decreased in si-β-catenin-treated SGC-7901 cells. Data are shown as the mean ± S.E.M, based on three to six independent experiments. **, p < 0.01

It has been reported that the Wnt signaling pathway can regulate the expression of miR-155. However, Whether MRTF-A is involved is unclear. To explore the potential relationship between miR-155 and Wnt signaling, we treated SGC-7901 cells with LiCl, an Wnt signaling pathway agonist, LiCl induced miR-155 expression, whereas MRTF-A knockdown significantly reversed the trend (Fig. 4a–c). To further examine the effects of Wnt signaling on miR-155 and MRTF-A expression, we treated SGC-7901 cells with Wnt3a, a specific ligand of the Wnt signaling pathway in SGC-7901 cells. The results showed that Wnt3a induced miR-155 expression whereas MRTF-A knockdown significantly reversed the trend (Fig. 4d–f). Next, β-catenin expression was depleted by specific siRNAs in SGC-7901 cells. As shown in Fig. 4g–i, β-catenin mRNA and protein levels were significantly decreased after siRNA transfection. In these β-catenin-depleted cells, MRTF-A and miR-155 levels were significantly decreased, indicating that β-catenin was required for miR-155 expression. These results indicate that Wnt/β-catenin regulates miR-155 expression through MRTF-A.

### miR-155 regulated GC migration and invasion by targeting the SOX1 gene

**Fig. 5:**
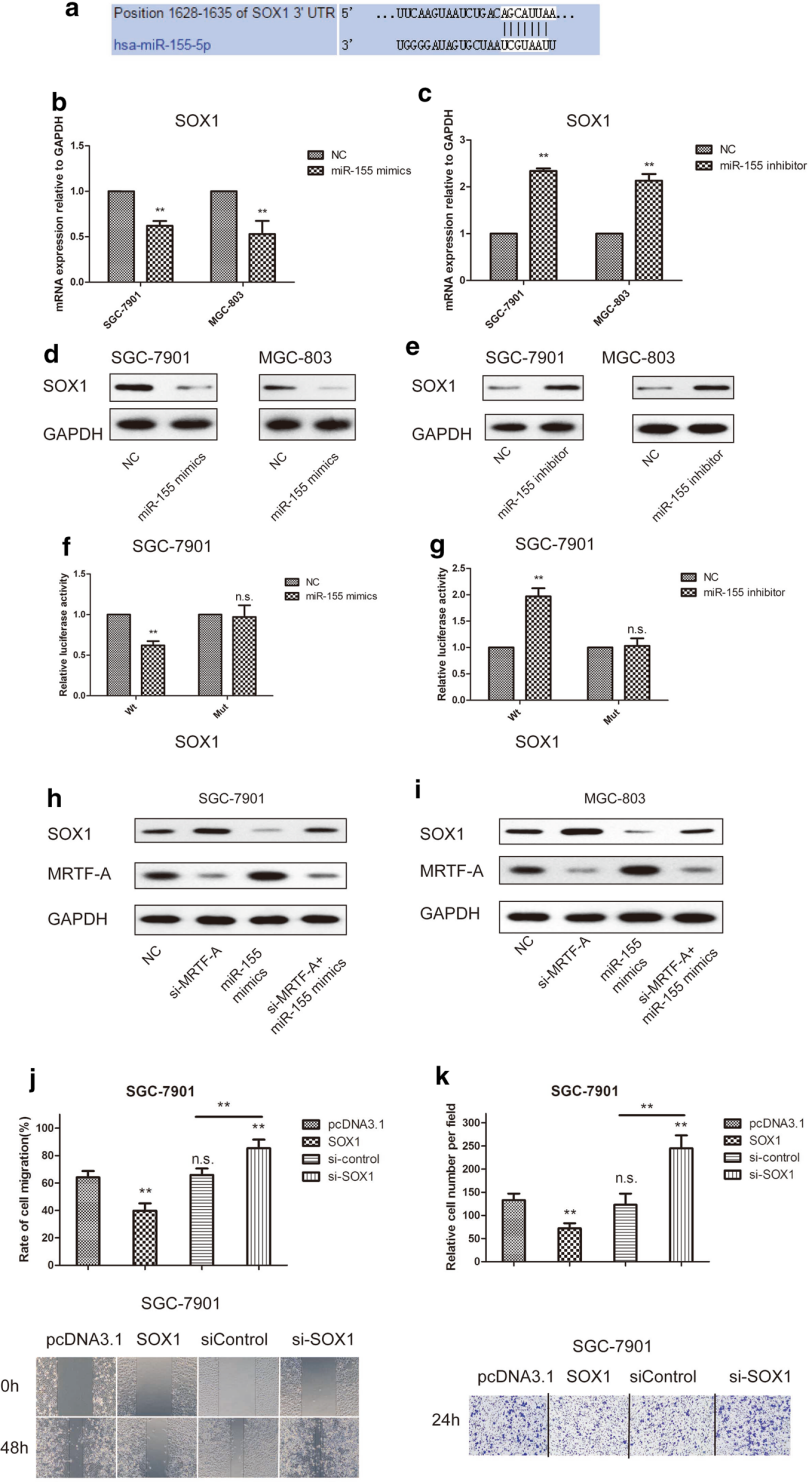
miR-155 regulated GC migration and invasion by targeting the SOX1 gene (**a**) Bioinformatics analysis of the possible binding sites between miR-155 and the SOX1 3′UTR. **b** miR-155 over-expression inhibited SOX1 RNA levels in SGC-7901 and MGC-803 cells. **d** miR-155 over-expression suppressed SOX1 protein levels in SGC-7901 and MGC-803 cells. miR-155 mimics were transfected into gastric cancer cells. Forty-eight hours after transfection, the mRNA levels were determined by RT-qPCR, and the protein levels were determined by Western-blot. GAPDH was used as a loading control. **c** miR-155 depletion with inhibitor upregulated SOX1 RNA levels in SGC-7901 and MGC-803 cells. **e** miR-155 depletion augmented SOX1 protein expression in SGC-7901 and MGC-803 cells. **f** miR-155 overexpression reduced SOX1 luciferase activity in SGC-7901 gastric cancer cells. **g** miR-155 depletion elevated SOX1 luciferase activity in SGC-7901 gastric cancer cells. **h**, **i** SOX1 and MRTF-A protein levels were determined by Western-blot in SGC-7901 cells and MGC-803 cells. **j** The effect of overexpressed or knocked down SOX1 on SGC-7901 migration of was detected by wound-healing assay. **k** Transwell assay was used to detect the migration-stimulating effects of overexpressed or knocked down SOX1 on SGC-7901. The number of cells that migrated to the lower side of the Transwell chamber was counted and photographed in five fields (the upper, lower, left, right, and middle fields) from three independent experiments, and the fold migration was calculated with the SPSS statistical software. Data are shown as the mean ± S.E.M based on three to six independent experiments. **, p < 0.01

miR-155 was identified as a potential regulator of SOX1 expression by miRNA target analysis using the Targetscan website. The predicted binding of miR-155 in the SOX1 3′UTR is shown (Fig. 5a). To determine the role of miR-155 on the SOX1 gene in gastric cancer cells, miR-155 mimics were transfected into SGC-7901 and MGC-803 gastric cancer cells. SOX1 mRNA and protein levels were significantly reduced followingmiR-155 overexpression (Fig. 5b, d). In contrast, SOX1 mRNA and protein levels were significantly upregulated after miR-155 inhibition (Fig. 5c, e). To confirm that SOX1 is a direct target of miR-155, a luciferase reporter assay was performed. The results indicated that co-transfection of cells with PGL3-SOX1 plus miR-155 mimics decreased luciferase activity, with no effects on the mutant (Mut) while co-transfection of cells with PGL3-SOX1 plus the miR-155 inhibitor significantly upregulated luciferase activity, without affecting the mutant (Mut) (Fig. 5f, g). These data suggested that SOX1 was a target of miR-155 in gastric cancer cells. We next examined whether MRTF-A regulated the SOX1 gene through miR-155 in gastric cancer cells, MRTF-A inhibition significantly suppressed the expression of SOX1. Co-treatment with miR-155 mimics significantly reversed these tumor-suppressive effects on gastric cancer cells (Fig. 5h, i). To determine the role of the SOX1 gene, the SOX1 gene was cloned and inhibited in SGC-7901 gastric cancer cells. Migration and invasion assays revealed that over-expression of SOX1 inhibited the migration and invasion of SGC-7901 cells (Fig. 5j, k). These results demonstrated that miR-155 regulated GC migration and invasion by targeting the SOX1 gene.

### MRTF-A regulated the expression of miR-155, which is related to migration and invasion

**Fig. 6:**
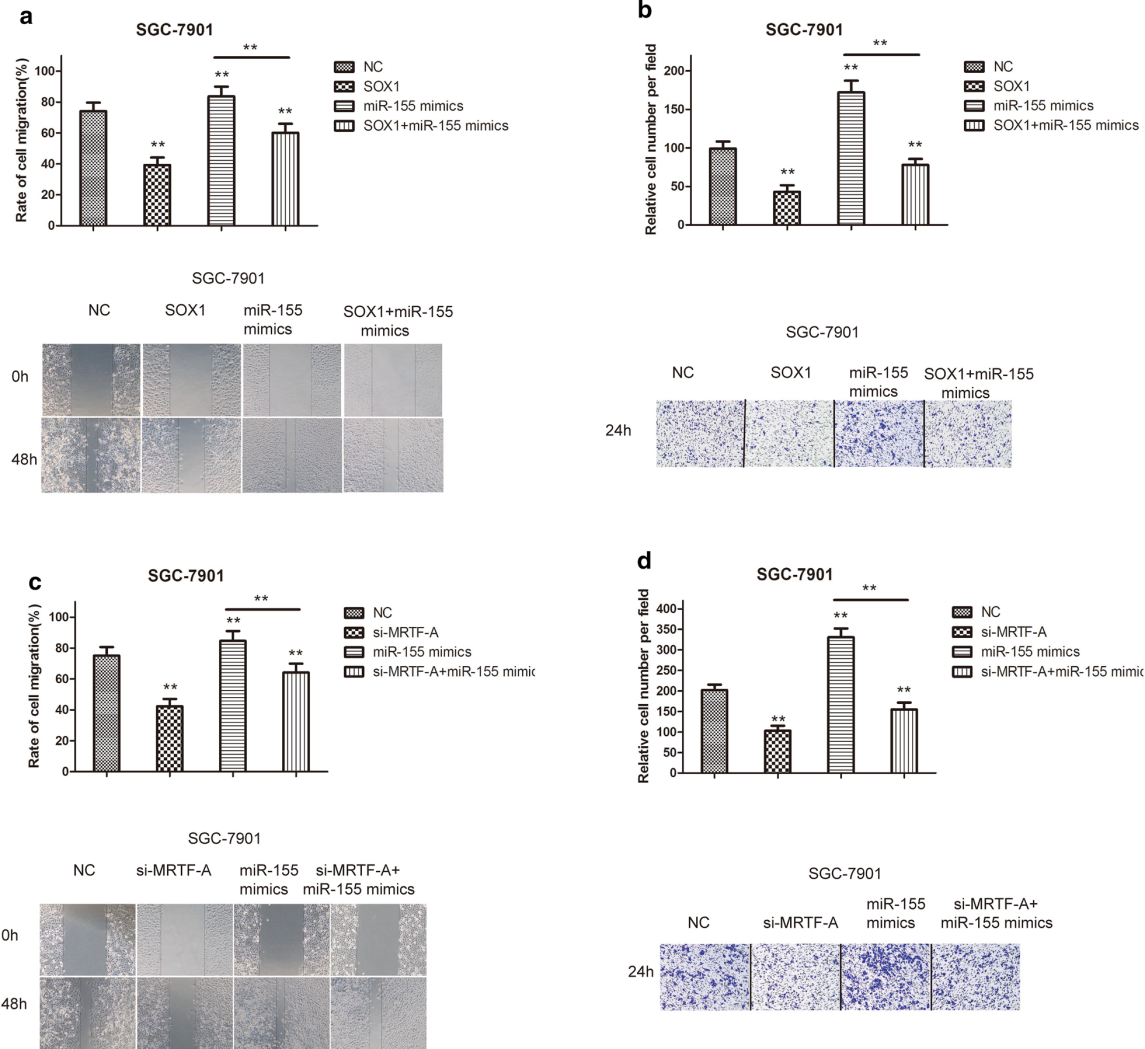
MRTF-A regulated the expression of miR-155, which is related to migration and invasion. **a**, **c** Results of scratch wound-healing experiments show the parallel migration of SGC-7901 cells. **b**, **d** Results of Transwell experiments show the transmembrane invasion of SGC-7901 cells. The number of cells that migrated to the lower side of the Transwell chambers was counted and photographed in five fields (the upper, lower, left, right, and middle fields) from three independent experiments, and the fold migration was calculated with the SPSS statistical software. Data are shown as the mean ± S.E.M based on three to six independent experiments. **, p < 0.01

To examine the influence of the miR-155 on SGC-7901 cell migration and the involvement of SOX1, migration and invasion assays were performed. The results showed that overexpression of SOX1 significantly suppressed the migration and invasion of gastric cancer cells. Co-treatment with miR-155 mimics significantly reversed the tumor-suppressive effects in SGC-7901 cells (Fig. 6a, b). We next sought to determine whether MRTF-A regulated the SOX1 gene through miR-155 in gastric cancer cells. Migration and invasion assays revealed that MRTF-A inhibition significantly suppressed the migration and invasion of gastric cancer cells, co-treatment with miR-155 mimics significantly reversed the tumor-suppressive effects on SGC-7901 cells (Fig. 6c, d), suggesting that miR-155 activity is required for MRTF-A-induced cell migration and invasion. Taken together, these findings indicated that MRTF-A regulated the expression of miR-155, which was related to migration and invasion.

### miR-155 downregulation inhibited gastric cancer growth in a nude mouse xenograft model

**Fig. 7:**
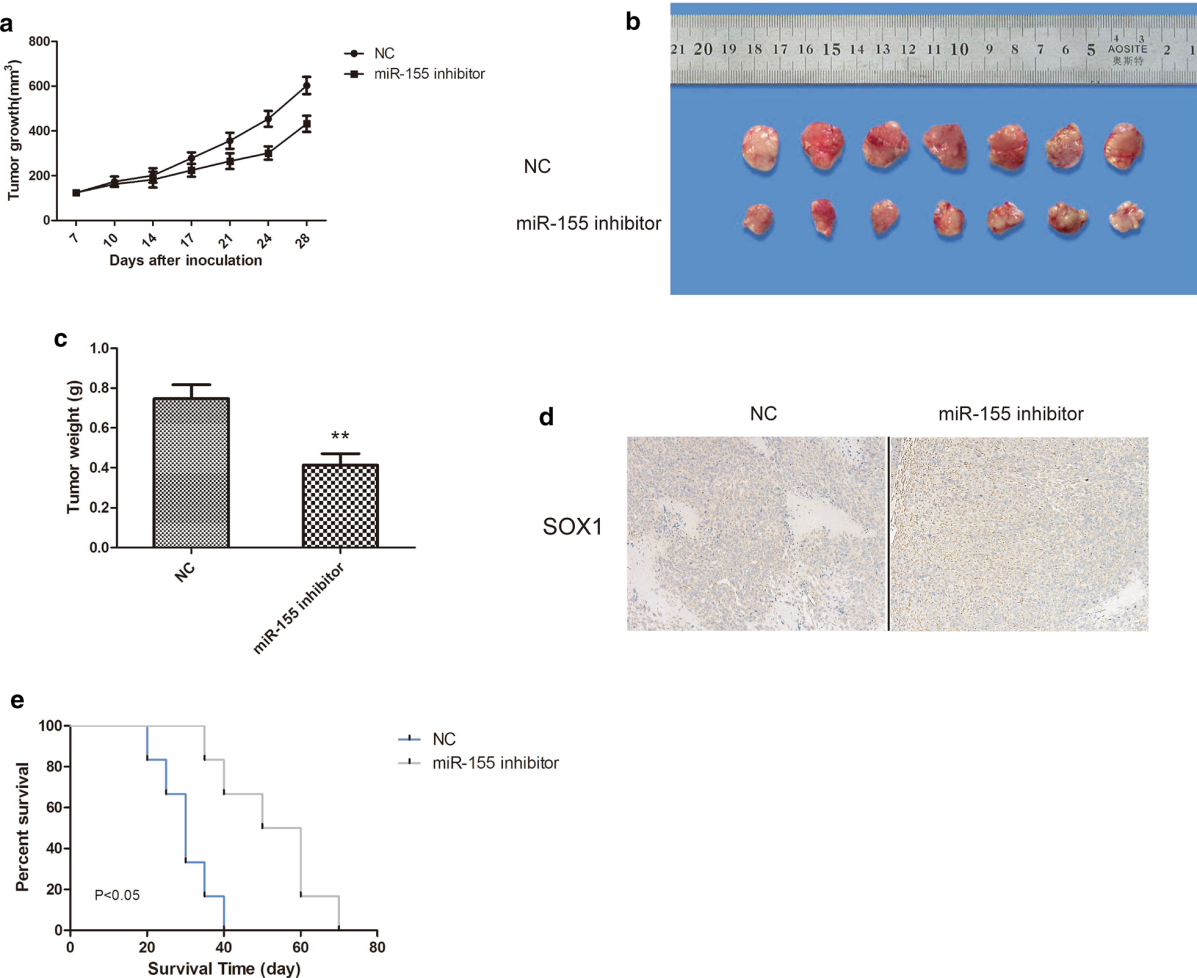
miR-155 overexpression promotes gastric cancer growth in a nude mouse xenograft model. **a** The tumor volume was calculated twice weekly. **b** Photographs of tumors derived from the NC and miR-155 inhibitor cells in nude mice. **c** Weights of tumors. **d** SOX1 expression was examined from SGC-7901 xenografts that were treated with or without miR-155 inhibitor-transfected cells. **e** Kaplan–Meier survival analysis of mice in different groups. Data are shown as the mean ± S.E.M based on three to six independent experiments. **, p < 0.01

We next examined whether inhibiting miR-155 in gastric cancer cells could decrease tumor growth in vivo. As shown in Fig. 7a, tumors formed from miR-155 inhibitor-transfected SGC-7901 cells grew significantly more slowly than those from the controls. The weights of the miR-155 inhibitor-treated tumors increased slower than the control tumors (Fig. 7b, c). Additionally, SOX1 expression was elevated after miR-155 inhibitor treatment of the xenograft tumors (Fig. 7d). Survival analysis revealed that down regulation of miR-155 in gastric cancer contributed to longer survival times (Fig. 7e). These data suggest that downregulation of miR-155 inhibited the development of gastric cancer.

## Discussion

It is generally accepted that the development of gastric cancer, such as other cancers, involves multiple steps, including the accumulation of genetic and epigenetic changes. In recent years, studies on epigenetic biomarkers such as miRNA expression have shown that they play an important role in the development of cancer. [28]. Thus, it is necessary to elucidate the role of cancer-specific miRNAs and their targets in tumor progression, as it is important for the design of novel therapeutic targets for cancer. [29–31]. Tumor migration is one of the most common phenomena in cancer. MRTF-A was previously identified as promoting EMT, a process that leads to fibrosis, during physiological wound-healing as well as in malignant and benign diseases. It has been reported that MRTF-A activity is associated with carcinogenesis. MRTF-A activity plays different roles in different cell types and tissue environments [32]. For example, inhibiting MRTF-A expression in breast cancer cells inhibited experimental metastasis in vivo as well as cellular motility [33, 34]. In the present study, we showed that MRTF-A played a positive role in the regulation of miR-155 expression in GC cells. MRTF-A physically associated with the miR-155 promoter to facilitate histone acetylation and RNAPolymerase II recruitment.

SOX1 is a member of the SOX transcription factor family and plays a crucial role in embryonic and postnatal development and stem cell regulation [17]. It has been reported that SOX1 can inhibit cervical, liver, and nasopharyngeal carcinomas through the Wnt/β-catenin signaling pathway [14]. Additionally, the SOX1 gene interferes with the STAT3 signaling pathway and regulates prostate cancer stem cell invasion [20, 35]. Our data showed that miR155 and SOX1 are inversely correlated. We found that miR-155 overexpression inhibited SOX1 luciferase activity. The results indicated that miR-155 regulated GC migration and invasion by targeting the SOX1 3′UTR region.

miR-155 is upregulated in cancer tissues and cell lines and promotes tumorigenesis and development [36, 37], including gastric cancer [38, 39]. It has been reported that miR-155 promoted the migration of colon cancer cells by targeting CBL [40]. However, its function and the relevant pathways with regards to SOX1 have not been sufficiently elucidated. In this study, we found an inverse correlation between miR-155 and SOX1 in GC cells. Subsequently, we found that miR‐155 directly bound to the SOX1 3′UTR. Furthermore, overexpression of miR-155 inhibited the expression of SOX1, while miR-155 inhibitors enhanced SOX1expression. The results illustrated that miR-155 acts as a direct upstream regulator of SOX1. Invasion and migration assays showed that elevated miR-155 expression or SOX1 silencing enhanced the cell invasion and migration abilities. Suppression of miR-155 expression or plasmid-mediated SOX1 overexpression inhibited invasion and migration in vitro. Additionally, suppression of miR-155 expression in vivo also inhibited tumor progression. Therefore, miR-155 acts an oncogene to directly regulate SOX1 expression and promote cancer progression in GC.

## Conclusion

In the present study, we identified a novel regulation mechanism which the MRTF-A/miR-155/SOX1 pathway mediates migration and invasion in GC.

## Data Availability

The datasets used or analysed during the current study are available from the corresponding author on reasonable request.
